# In-vivo assessment of retinal vessel diameters and observer variability in mice: A methodological approach

**DOI:** 10.1371/journal.pone.0271815

**Published:** 2022-07-21

**Authors:** Lukas Streese, Jeannine Liffert, Walthard Vilser, Christoph Handschin, Henner Hanssen

**Affiliations:** 1 Department of Sport, Exercise and Health, Medical Faculty, University of Basel, Basel, Switzerland; 2 Biozentrum, Medical Faculty, University of Basel, Basel, Switzerland; 3 Institute of Biomedical Engineering and Informatics, Technical University of Ilmenau, Ilmenau, Germany; INSERM, Université de Bordeaux, FRANCE

## Abstract

**Background:**

Central retinal arteriolar (CRAE) and venular (CRVE) diameter equivalents are predictive for cardiovascular and all-cause mortality in humans. The aim of this study was to investigate the inter- and intraobserver variability for the assessment of CRAE and CRVE in mice using fluorescein contrast enhancement as compared to crude analysis.

**Methods:**

Three high quality images with (F) and without fluorescein (NF) of eight mice (type C57BL) were recorded and analysed by two independent experienced investigators to investigate interobserver variability. In addition, one investigator analysed 20 F and 20 NF images twice to investigate intraobserver variability. The time course of CRAE and CRVE vessel responses after fluorescein injection were recorded in one mouse every 30 seconds for 15 minutes.

**Results:**

The interobserver variability was lower in F images compared to NF images for CRAE (r = 0.99, p < 0.001 vs. r = 0.65, p = 0.083) and CRVE (r = 0.99, p < 0.001 vs. r = 0.79, p = 0.019). Intraobserver variability for CRAE (r = 0.99, p < 0.001 vs. r = 0.48, p = 0.032) and CRVE (r = 0.98, p < 0.001 vs. r = 0.86, p < 0.001) were lower in F compared to NF images. Fluorescein injection induced vascular staining mimicking vessel dilation (+14%) followed by a long-lasting stable staining phase well suited for precise measurements.

**Conclusions:**

Measurement variability can be optimized by use of fluorescein as contrast enhancement in mice. Standardization for time of image acquisition after fluorescein injection is advisable. Translation of static retinal vessel analysis into a rodent model has the potential to bridge the research gap between proof of concept studies in animals and clinical studies in humans.

## Introduction

Cardiovascular (CV) diseases are still the main cause of non-communicable diseases worldwide [1]. Central retinal arteriolar (CRAE) and venular diameter equivalence (CRVE) are sensitive and non-invasive, in-vivo microvascular biomarkers to detect systemic CV risk in humans. Narrower CRAE and wider CRVE are associated with higher CV risk such as high blood pressure [2, 3], inflammation [4] or stiff arteries [3], as well as manifest CV diseases like coronary heart disease [5] and stroke [6] as well as CV mortality [7]. Assessment of CRAE and CRVE in addition to classical CV risk factors improved CV risk stratification up to 20% in humans [8, 9]. We have recently published standard operating procedures as well as normative data for the assessment of CRAE and CRVE in humans [10]. The transfer of the well-established static retinal vessel analysis in humans into mouse models would allow for bridging the research gap between proof of concept studies in animals and clinical studies in humans.

Mouse models are used to study cause and effect of treatment options and to give mechanistic insights on how to treat and prevent non-communicable diseases in humans. Cardiovascular physiology and pathophysiology in mice have several common traits as compared to humans [11]. Few studies have previously investigated retinal vessel diameters as a biomarker of systemic microvascular health in mice [12, 13]. However, no study to date investigated the standardized method to quantify CRAE and CRVE in mice by use of in-vivo static retinal vessel analysis, a method routinely applied in humans.

This study aimed to investigate inter- and intraobserver variability of CRAE and CRVE with and without fluorescein injection in mice as a basis for further validation in mouse models of CV disease and to allow for precision research in a mouse model of retinal microvascular health. In addition, this study aimed to quantify the time course of CRAE and CRVE vessel responses after fluorescein injection.

## Material and methods

### Study design

This study was conducted in accordance to the ARRIVE guidelines. Investigations took place in the Animal Laboratory at the Biozentrum of the University of Basel. Image analyses were performed at the Department for Sport, Exercise and Health of the University of Basel. All procedures involving animals were approved by the veterinary office of the canton Basel-Stadt/Switzerland (approval no. 2329) and institutional authorities, and performed in accordance with the Swiss federal guidelines for animal experimentation under consideration of the wellbeing of the animals and the 3R (replace, reduce, and refine) principle. Not specific exclusion criteria were defined.

### Mouse preparation

Eight male mice (C57BL/6JRj) aged 15 weeks were anesthetised with isoflurane (1.5%) by supplementing nitrous oxide as recommended previously [14] using flow-controlled inhalation. 0.5% tropicamide was used to dilate one pupil. The cornea was locally anesthetised with 4mg/ml EDO^®^ eye drop solution. The hard mouse contact lenses (CANTOR+NISSEL^®^) prevented the eye from drying out to fulfil the optical requirements for the fundus images. The other eye was treated with one drop of Methocel 2% (OmniVision^®^) to protect the eye. Vaseline was used to make the whiskers stick to the fur, in order to prevent adulteration of the fundus images. Fluorescein (25% fluorescein sodium) was injected with a concentration of 0.4 μl/gram body weight subcutaneously into the nuchal fold. Mice were placed in a 65-degree angle about 2cm in front of the lens on a heated map to conduct retinal vessel imaging at focus. Finally, sacrifice was performed following institutional and federal guidelines using flow-controlled CO_2_ inhalation and ascertained by cardiac puncture and blood removal. Score sheets were used to record abnormalities and suffering following the criteria established and approved in the animal protocol. If accumulated scores exceeded a pre-defined threshold, mice were sacrificed and removed from the experiment.

### Assessment of retinal vessel diameters

**Fig 1:**
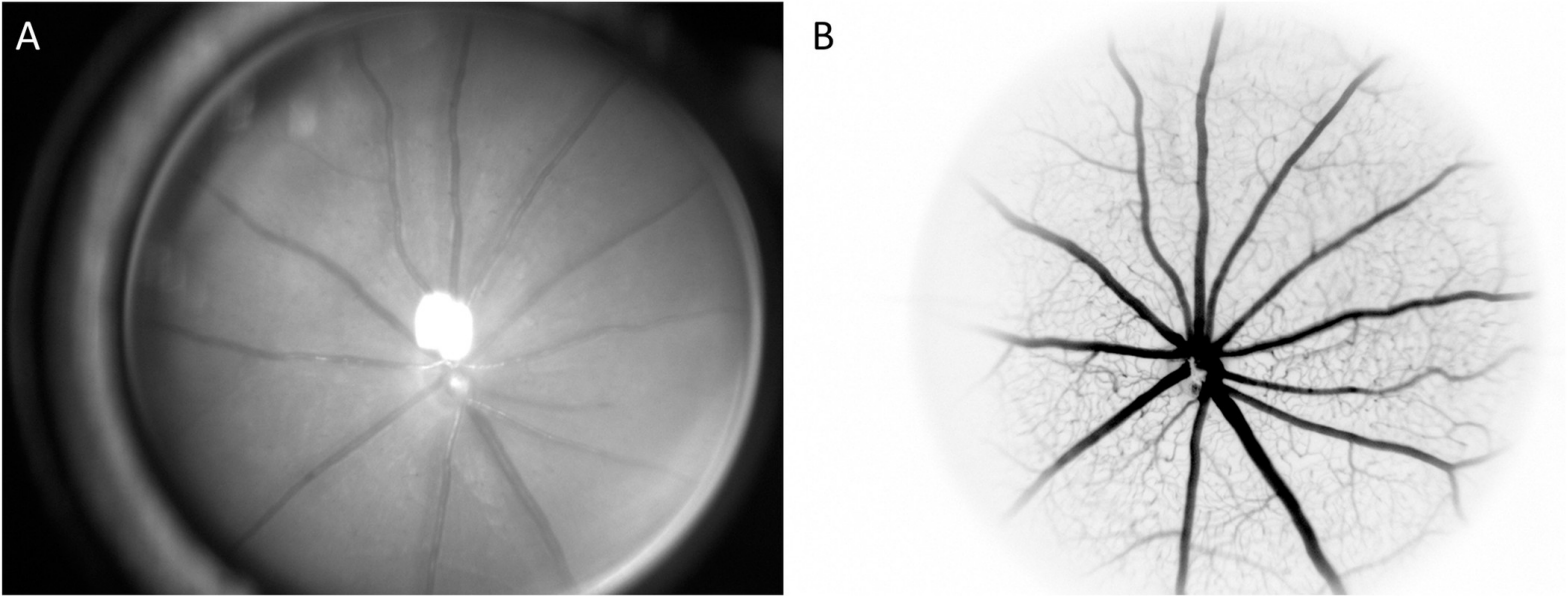
Fundus images of one mouse without and with fluorescein. Retinal vessels of one mouse without (A) and with fluorescein (B).

**Fig 2:**
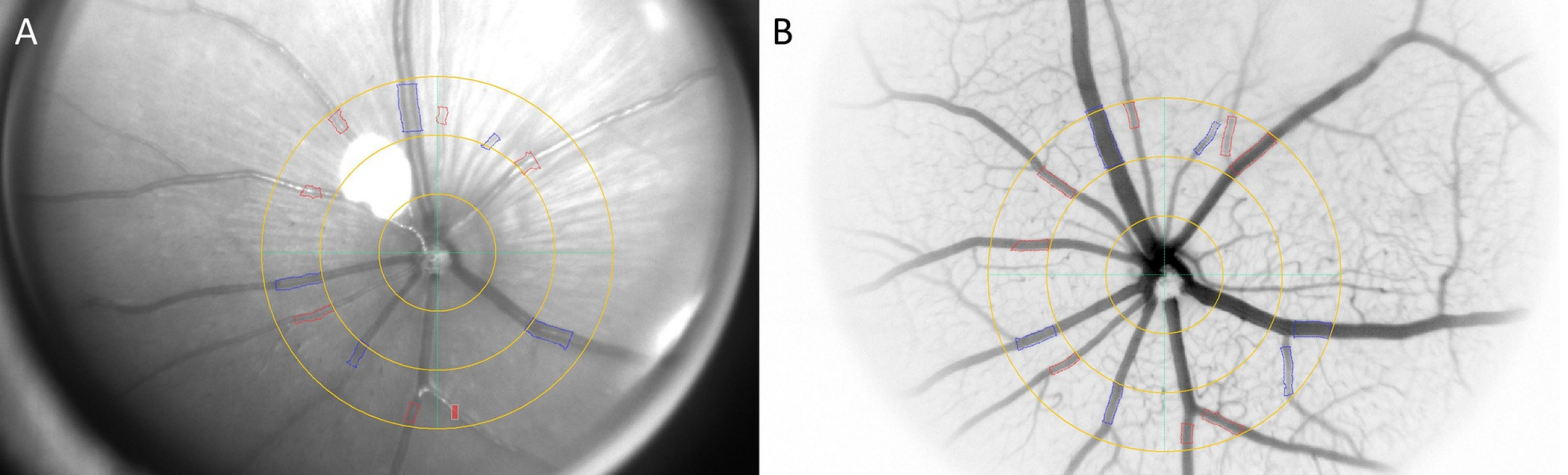
Fundus images with marked vessel segments of one mouse without and with fluorescein. Retinal vessels of one mouse without (A) and with fluorescein (B) with marked arteriolar (red) and venular (blue) vessel segments. Three images of eight mice with and three images of eight mice without fluorescein were averaged by investigator one and two separately to investigate interobserver variability. Twenty images without and 20 images with fluorescein were analysed by one investigator twice to investigate intraobserver variability.

The RCrodent FAG system (Imedos Systems GmbH, Jena, Germany) was used to non-invasively investigate retinal microvascular diameters by static retinal vessel analysis. Three high-quality images without (NF) and afterwards with fluorescein (F) of eight mice were taken. Fig 1 shows the difference between NF (Fig 1A) and F images (Fig 1B). In one of these eight mice, images were taken every minute after fluorescein injection to investigate the time course of fluorescein-induced retinal vessel diameter responses until 15 minutes after the injection. Two experienced investigators analysed the same three images with and without fluorescein individually as well as images describing the time course after fluorescein injection by using a specified analysing software (Vesselmap 2^®^, Imedos Systems GmbH, Jena, Germany). The analysing procedure was equivalent to the procedure used in humans and described in detail previously [10]. Briefly, digital images of one eye were taken by use of a rodent retinal vessel analyser attached to a charged-couples camera device (Imedos Systems GmbH, Jena, Germany). Diameters of retinal arterioles and venules were analysed semi-automatically at higher magnification in an area of 0.5–1 disc-diameter from the optic disc margin using the above analysing software. Retinal arterioles and venules were detected and measured in the outer ring zone as demonstrated in Fig 2. Diameters were averaged to central retinal arteriolar (CRAE) and central retinal venular (CRVE) equivalents using the Paar-Hubbard formula [15].

Both investigators semi-automatically marked arteriolar and venular vessel segments. The analysing software calculated CRAE and CRVE in measuring units based on these vessel segment. CRAE and CRVE were averaged based on three F and three NF images per mouse for investigator one and two separately to investigate interobserver variability

One investigator analysed CRAE and CRVE of 20 F and 20 NF images a second time to investigate intraobserver variability. Do to the fact that F and NF images are very different (Fig 1), blinding of investigators for image condition was not possible. However, investigators were blinded for the timepoint of image acquisition during the analytical process.

### Statistical analysis

The average of CRAE (primary outcome) and CRVE (secondary outcome) of three F and NF images were calculated for every mouse and investigator separately. Two-tailed Pearson correlations were computed in F and NF images separately to analyse the interobserver variability between investigator 1 and 2 for CRAE and CRVE. The coefficient of variation for CRAE and CRVE was analysed for F and NF images as well as for investigator 1 and 2 separately. The intraobserver variability was analysed by calculating two-tailed Pearson correlations as well as the coefficient of variation of CRAE and CRVE separately. Images that described the time course were analysed by two experienced investigators separately and were thus averaged. R version 3.4.2 for Windows was used for data analysis and scatterplots. The level of significance was set at p < 0.05. No animal was excluded from the analysis.

## Results

### Interobserver variability

**Fig 3:**
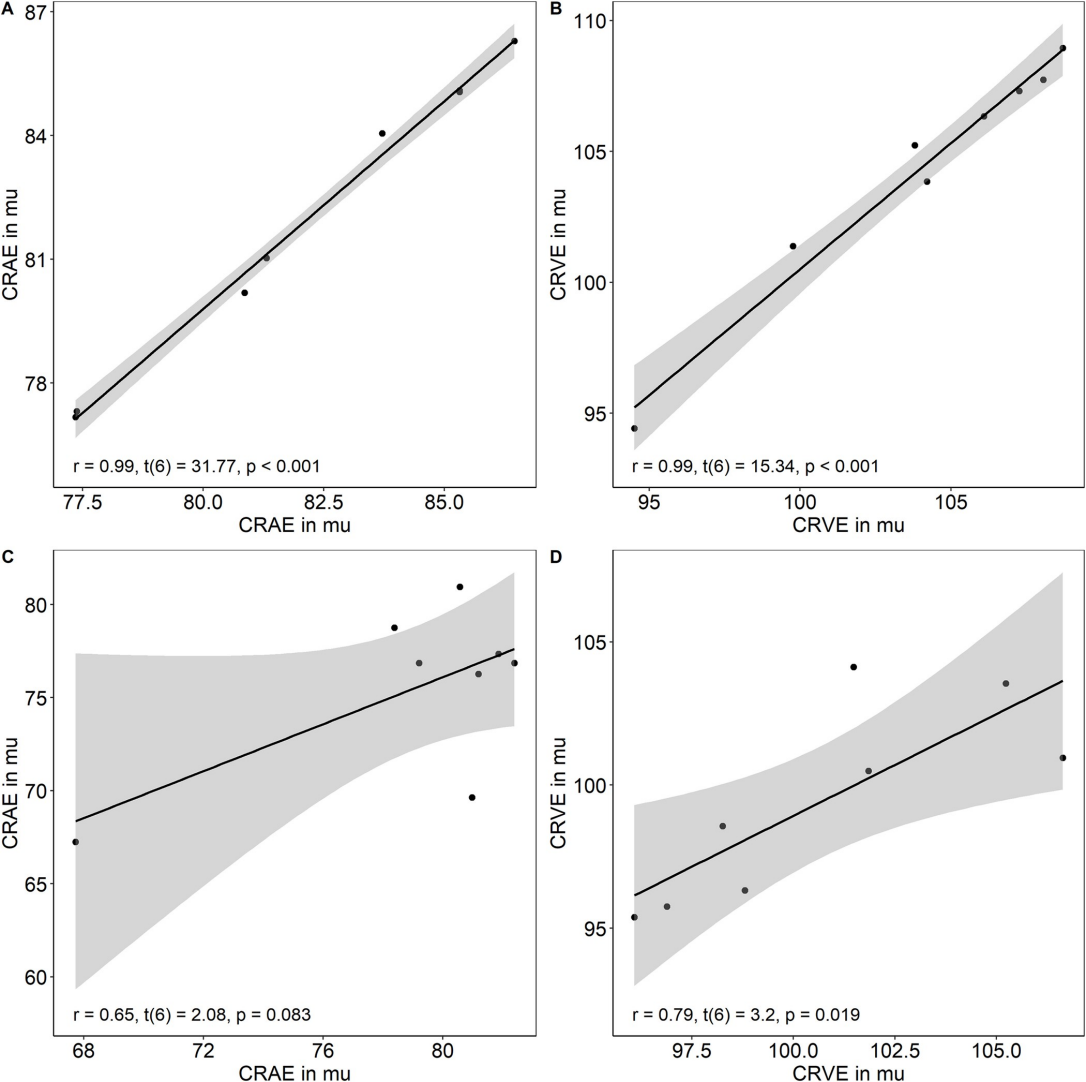
Interobserver variability. Interobserver variability of central retinal arteriolar (CRAE) and venular diameter equivalents (CRVE) were analysed. Three images from eight mice with (A and B) and three images from the same eight mice without fluorescein (C and D) were averaged and analysed by investigator one and two separately to investigate interobserver variability. Two-tailed Pearson correlations were computed in fluorescein and none-fluorescein images separately to analyse the interobserver variability between investigator one and two for CRAE and CRVE separately.

Eight 15 weeks old male mice (C57BL/6JRj) with a mean body mass of 32.2±1.3g were analysed to investigate inter- and intraobserver variability. F images showed lower interobserver variability for CRAE (r = 0.99, t(6) = 31.77, p < 0.001 vs. r = 0.65, t(6) = 2.08, p = 0.083) and CRVE (r = 0.99, t(6) = 15.34, p < 0.001 vs. r = 0.79, t(6) = 3.2, p = 0.019) compared to NF images (Fig 3). The coefficient of variation ranged between 4.3% and 4.6% in F and between 3.5% and 6.1% in NF images.

### Intraobserver variability

**Fig 4:**
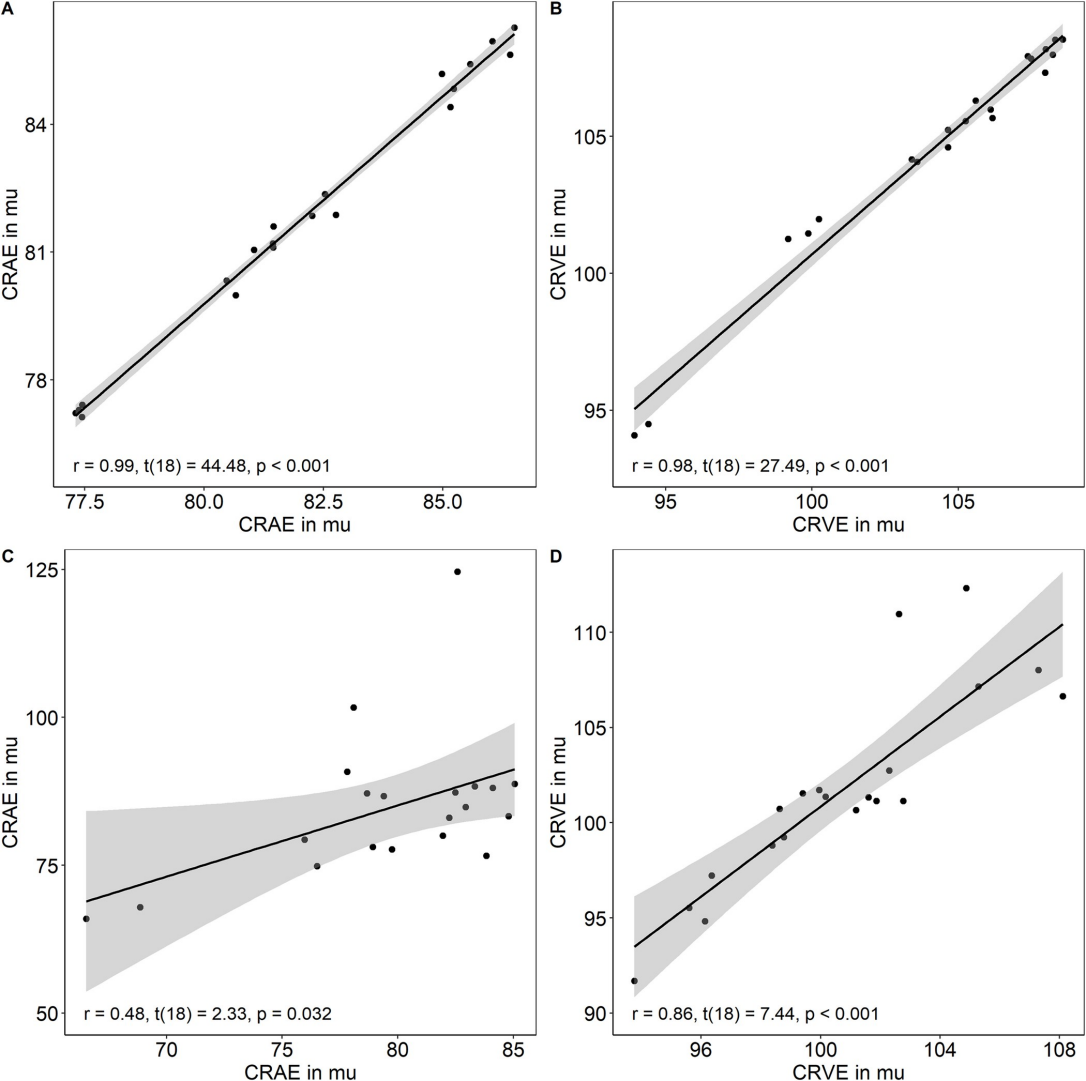
Intraobserver variability. Twenty images with (A and B) and 20 images without fluorescein (C and D) were analysed twice by one investigator to quantify intraobserver variability. Images were randomly selected. The intraobserver variability was analysed by calculating two-tailed Pearson correlations as well as the coefficient of variation of CRAE and CRVE separately.

Intraobserver variability for CRAE (r = 0.99, t(18) = 44.48, p < 0.001 vs. r = 0.48, t(18) = 2.33, p = 0.032) and CRVE (r = 0.98, t(18) = 27.49, p < 0.001 vs. r = 0.86, t(18) = 7.44, p < 0.001) were lower by analysing F images compared to NF images (Fig 4). The coefficient of variation of 20 images analysed by the same investigator on two different evaluation timepoints ranged between 3.6% and 4.3% in F and between 3.8% and 14.6% in NF images.

### Time course after fluorescein injection

**Fig 5:**
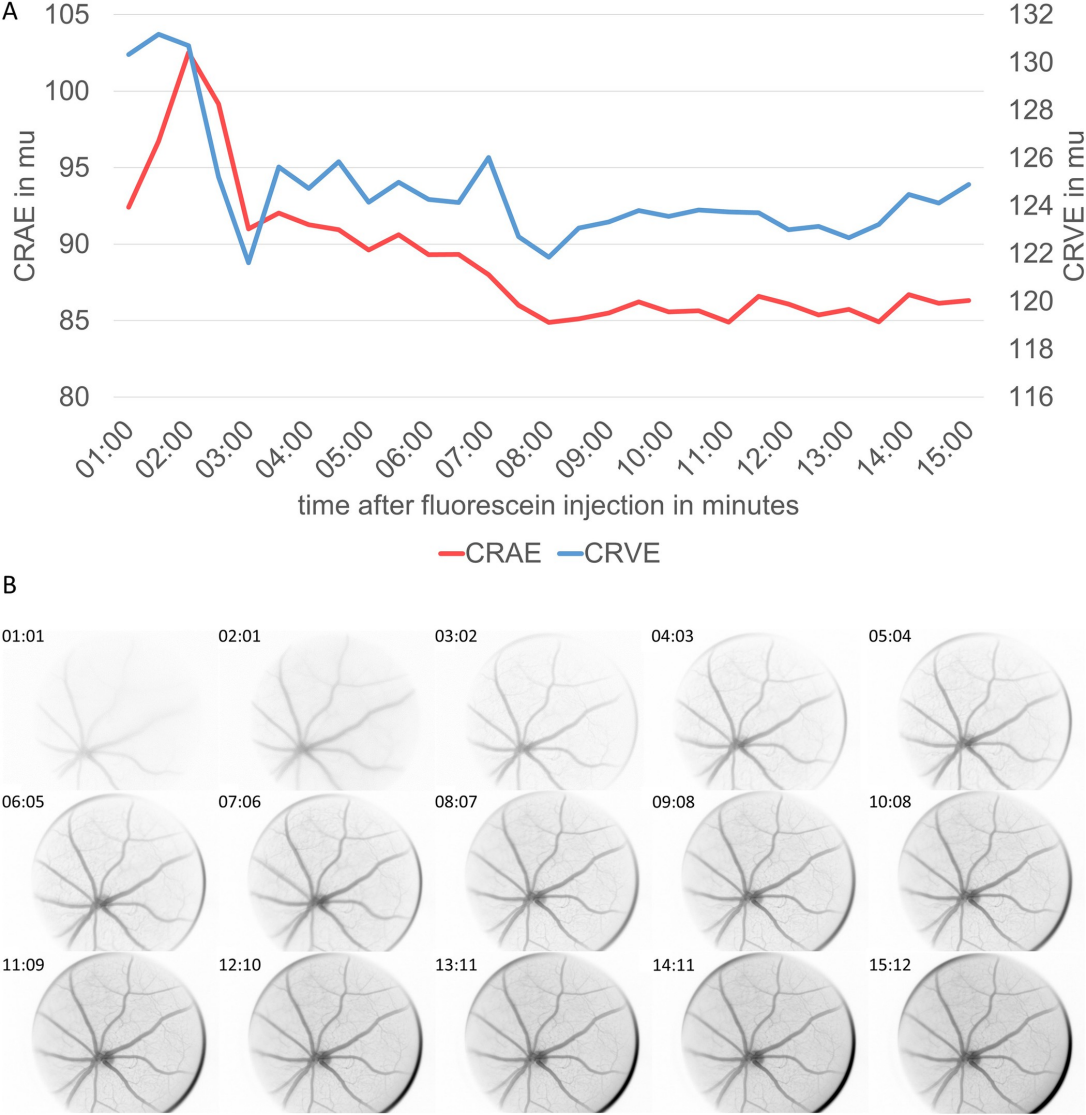
Time course after fluorescein injection. Time course of central retinal arteriolar (CRAE) and venular diameter equivalents (CRVE) after fluorescein injection in one mouse (A). CRAE and CRVE were averaged based on one image per timepoint analysed independently by two experienced investigators. Results are presented descriptively. Fundus images of one mouse every minute after fluorescein injection until 15 minutes after injection (B).

Fluorescein injection lead to a fast vessel staining (Fig 5) that influenced CRAE and CRVE directly after the injection. Arteriolar equivalents seemed to be wider (+14%) with its peak 2:30 minutes after the injection followed by a constant diameter decline till eight minutes after injection, when the baseline was reached and no further diameter variations occurred (Fig 5).

## Discussion

Static retinal vessel imaging in mice is feasible and variability of vessel diameters can be minimized by use of fluorescein as contrast enhancement reducing both inter- and intraobserver variability of arteriolar and venular diameters. Timing of retinal vessel image acquisition and analysis is essential to allow for stable and precise diameter assessments 8 to 10 minutes after fluorescein injection.

Non-invasive and in-vivo retinal vessel analysis facilitates the investigation of microvascular health in mice allowing to quantify intervention effects of treatment strategies and mechanisms of change in the microvascular bed. We have recently demonstrated that retinal vessel diameters reflect systemic CV risk [16, 17], associate with mechanistic pathways [17, 18] and are sensitive microvascular biomarkers to quantify treatment effects in humans [19, 20]. Most other microvascular techniques necessitate post-mortem analyses in mouse models. Retinal vessel analysis has the unique advantage of allowing analysis in the arteriolar and venular microvascular bed separately, over a longer period of time and in the same living mouse. However, high image quality is essential to avoid measuring errors and allow for reduced data variability, a prerequisite for high reproducibility and validity.

Fluorescein injection for contrast enhancement provided a clearer delineation of blood vessels and peripheral tissues compared to NF images. Not all retinal vessels could be clearly differentiated form surrounding tissue in crude NF images, even in images with the best quality. The same phenomenon has been reported previously [21]. In addition, arterioles often present with a flashlight reflection alongside its longitudinal axis, which adds to the challenge of differentiation from underlying neuronal striae-like fibres. Oftentimes, the semi-automated software was not able to detect the arteriolar diameters and manual reanalysis had to be performed adding to the higher variability in the crude approach without fluorescein enhancement.

The intravenous injection of 25% fluorescein sodium induced an early dynamic phase of vessel diameters after injection. This effect is mainly caused by the dynamic colouring of the first inflowing dye. In this phase, the streaming blood column in the vessels is stained differently and the stained parts of the blood flow are not representative for the vascular lumen, which results in massive measurement errors. This is well known from human fluorescence angiography. For this reason, measurements should only be carried out in the stable phase that follows after 8 minutes, which enables precise measurements over a longer period of time until the coloration begins to decrease.

Our results demonstrate the importance to standardize the timepoint of image acquisition to avoid possible measuring fluorescein-induced diameter variations when assessing CRAE and CRVE as outcome variables in mice. We recommend image acquisition at least 8 to 10 minutes after fluorescein injection. Eight minutes after the injection the fluorescein induced diameter variations have dissolved with stable diameters in contrast enhanced retinal vessels (Fig 5A and 5B). Fluorescein enhancement improves image quality with clearer contrast patterns allowing for precision analysis as a necessary precondition for high reproducibility and validity. In fact, it may be advisable for each laboratory and for each study set up to individually define the optimal time of image acquisition as performed in this study in a single example.

### Strength and limitations

Our study results demonstrate the feasibility of producing high quality retinal in-vivo images from mice with lower variability and higher precision following fluorescein contrast enhancement. Here, we focused on feasibility of fluorescein use and assessment of observer variability. Future studies will have to assess reproducibility on separate days as well as validity to differentiate vessel diameters in healthy mice versus mouse models of CV disease. It is important to note that the choice of anesthetic needs careful consideration as different anesthetics have been shown to have an impact on functional retinal blood flow in the rat eye [22] and are thus likely to also affect retinal vessel diameter. In our study we have used isoflurane and we cannot rule out that it affected the assessment of retinal vessel diameters to some extent.

Moreover, the mice in our study were all male and 15 weeks of age. Future research is warranted to confirm feasibility and low intra- and interobserver variability in mice of different age and gender. This is a methodological proof of concept study and further research will have to be performed in mutant knock-out mouse models of CV disease in order for the mouse model to give more thorough mechanistic insights and to potentially be used for development of new therapeutic targets for the treatment of CV disease.

### Conclusions

The precision of static retinal vessel imaging in a mouse model can be optimized by application of fluorescein as a contrast enhancer, whereby intra- as well as interobserver variability are minimized. We further identified the importance for timing of image acquisition aiming for data analysis, in our example, no earlier than 8–10 minutes after subcutaneous fluorescein administration. Repeated time course analyses for optimal timing of image acquisition after fluorescein application is advisable as a first step before the start of main data acquisition as way of calibration of the experimental set up. Our study results are prerequisites to enable validation of retinal vessel diameters in mouse models of CV diseases, including randomized controlled intervention trials. Application of retinal vessel analysis in rodent models using the same principle in-vivo approach as applied in humans allows to bridge the translational research gap between “bench-side” proof of concept studies in animals and “bedside” clinical studies in humans. The unique approach will also allow for new frontiers, for example, in pharmaceutical research as mechanisms and microvascular effects of new drugs, such as antihypertensive, antidiabetic or lipid-lowering agents can be examined from basic animal models to clinical studies throughout all phases of drug development.

## Supporting information

S1 Checklist ARRIVE checklist. (PDF)

S1 Dataset Interobserver variability analysis. (XLSX)

S2 Dataset Intraobserver variability analysis. (XLSX)

S3 Dataset Timeline analysis. (XLSX)
